# Cost consequences of unscheduled emergency admissions in cancer patients in the last year of life

**DOI:** 10.1007/s00520-023-07633-6

**Published:** 2023-03-04

**Authors:** Ethna McFerran, Victoria Cairnduff, Ray Elder, Anna Gavin, Mark Lawler

**Affiliations:** 1 C/o Patrick G Johnson Centre for Cancer Research, Queen’s University Belfast, 97 Lisburn Road, Belfast, BT9 7AE UK; 2 Centre for Public Health, Queen’s University Belfast, Belfast, UK; 3 South Eastern Health and Social Care Trust, Ulster Hospital, Upper Newtownards Road, Dundonald, BT16 1RH UK; 4 Northern Ireland Cancer Registry, Mulhouse Building, Queen’s University, Mulhouse Rd, Belfast, BT12 6DP UK; 5 School of Medicine, Dentistry and Biomedical Sciences, Queen’s University Belfast, 97 Lisburn Road, Belfast, BT9 7AE UK

**Keywords:** Neoplasms, Death, Palliative care, Emergency care, Cost consequences

## Abstract

**Objectives:**

Cancer is a leading cause of death. This paper examines the utilisation of unscheduled emergency end-of-life healthcare and estimates expenditure in this domain. We explore care patterns and quantify the likely benefits from service reconfigurations which may influence rates of hospital admission and deaths.

**Methods:**

Using prevalence-based retrospective data from the Northern Ireland General Registrar’s Office linked by cancer diagnosis to Patient Administration episode data for unscheduled emergency care (1st January 2014 to 31st December 2015), we estimate unscheduled-emergency-care costs in the last year of life. We model potential resources released by reductions in length-of-stay for cancer patients. Linear regression examined patient characteristics affecting length of stay.

**Results:**

A total of 3134 cancer patients used 60,746 days of unscheduled emergency care (average 19.5 days). Of these, 48.9% had ≥1 admission during their last 28 days of life. Total estimated cost was £28,684,261, averaging £9200 per person. Lung cancer patients had the highest proportion of admissions (23.2%, mean length of stay = 17.9 days, mean cost=£7224). The highest service use and total cost was in those diagnosed at stage IV (38.4%), who required 22,099 days of care, costing £9,629,014. Palliative care support, identified in 25.5% of patients, contributed £1,322,328. A 3-day reduction in the mean length of stay with a 10% reduction in admissions, could reduce costs by £7.37 million. Regression analyses explained 41% of length-of-stay variability.

**Conclusions:**

The cost burden from unscheduled care use in the last year of life of cancer patients is significant. Opportunities to prioritise service reconfiguration for high-costing users emphasized lung and colorectal cancers as offering the greatest potential to influence outcomes.

## Introduction

Cancer is a leading cause of death worldwide [1]. Whilst ‘death and dying are inevitable’ [2], the moments leading to death create the causes and conditions for the development of meaning, thereby constructing a peaceful or traumatic transition. Palliative care underpins symptom management in the end of life and is considered a human right [3], since its absence can lead to unnecessary suffering [4]. Given the rise in cancer incidence in coming decades [5], and the fact that end-of-life expenditure and utilisation of healthcare services are peak [6, 7] (up to 20% of NHS budgets [8]), it is essential to examine how patients can receive care throughout their cancer pathway, into palliation.

Improving outcomes during end of life is grounded in patients’ use of and experiences in healthcare services. Realising preferences for home-based [9] and individualised end-of-life care are recommended [10]. Discussions of preferred place of death support the management of care complexity [11, 12]. However, difficulties finding information on advanced planning care [13], potentially exacerbate dissonance between stated and revealed preferences and lead to a significant proportion of patients dying in hospital [14, 15].

Those approaching end of life should expect coordinated care [16]; however, service gaps can lead to emergency hospital admissions which are not always necessary or medically indicated [17]. Such admissions alongside other quality indicators (*short interval between chemotherapy and death, high proportion of hospital deaths vs home deaths, frequent emergency room visits, high number of hospital/Intensive Care Unit days near the end of life and hospital admission near to death*) can identify healthcare systems delivering aggressive or poor-quality care [18]*.* Furthermore, readmissions believed undesirable are associated with care quality during initial hospital stays, transitional care and post-discharge support [19]. Consequently, 30-day readmission rates and admissions in the last 28 days of life may be meaningful metrics in examining coordination issues. Conversely, indicators of good-quality care, communication, shared decision-making, advance directives and pain and symptom management [18], are infrequently available within patient datasets to examine end-of-life experiences.

Palliative care reduces symptom burden, improves satisfaction and quality of life of patients and caregivers and potentially extends the length of life [20]. Economic studies addressing palliative care suggest savings result from fewer hospitalisations and reduced use of acute care. Notably, hospital palliative care input has been associated with a reduction in readmission [21].

Palliative care access and ‘in-home’ procedures may prevent emergency admissions given the symbiotic relationship between primary and tertiary care [17]. The appropriate method for controlling symptoms in some patients will be hospital-based; however, the difference between hospital visits indicating necessary care versus aggressive care varies [22]. Therefore, to target and scale palliative care service development and identify those likely to benefit from service improvement, critical data insights are needed.

Thus, we evaluate the cost characteristics of a Macmillan-funded report [15] commissioned to examine unscheduled care use before the introduction of acute oncology services in 2016. This study examines the length of stay (LOS) costs and assesses for potential predictors of LOS in cancer patients in Northern Ireland (NI). Study results support the prioritisation of services, delivered in the right place, to the right patient, which may reduce avoidable emergency admissions and hospital-based deaths.

## Methods

We conducted a retrospective cost consequence study using a prevalence-based approach by secondary data analysis, including all patients who died in 2015 in NI of cancer (ICD10 C00-C97) and report in line with guidelines [23]. Within the secure environment of the Northern Ireland Cancer Registry, anonymised data extracted from the NI General Registrar’s Office was linked (by cancer diagnosis) to Patient Administration System episodes for unscheduled emergency care (UEC) admissions between 1st January 2014 and 31st December 2015; these data were collected to capture all UEC episodes experienced in the last year of life for each cancer patient to examine patterns of how unscheduled care services were being used before the introduction of acute oncology teams.

In the initial report of this data [15], there were higher rates of emergency admissions for haematological, brain and CNS, lung and mesothelioma cancers. Infection was cited as the most common cause of admissions (23.9%). Of those admitted for emergency care in the last year of life, 10.8% had been diagnosed with cancer on the same day. Of the emergency admissions, 33.4% took place during normal working hours. The report provided a limited examination of LOS, identifying that 1 in 5 (20.5%) had a total inpatient stay of more than 28 days.

The primary aim of our study was to calculate a cost scenario by the LOS outcomes, from the payer perspective [24]. Since these anonymised data provided diagnostic codes but limited detail of specific care performed during admission, micro-costing was not possible. Additional data mining provided codes to cost palliative care support and GP/ambulance involvement for each admission.

Clinical expert input verified that a direct palliative care admission was not standard practice during the data extract period; therefore, in line with costing recommendations, costs were included per bed day [25]. All calculations are based on ‘in-year’ (2015) estimated national average costs, shown in Table 1. Analyses were conducted in ‘R’.

**Table 1:** Cost assumptions

Variable	Cost (£)	Description
Non-elective inpatient (excluding excess bed days)	1609	1609/day of admission [24]
Excess bed day (day 2 onwards)	306	+ 306 per excess bed day [24]
Inpatient – specialist palliative care (support) cost per day	119	If a patient is not admitted under the care of a specialist palliative medicine consultant but is receiving support from a member of a specialist palliative care team, this is classed as specialist palliative care support and should be reported per bed day [25].
Ambulance transfer [24]	236	Based on reports where admissions via ambulance or paramedic admission were reported*, they were assumed to have received a ‘see, treat and convey’ cost per episode.
GP referral	39.23	Where patient-level records reported an admission method as ‘GP via ambulance’, the additional cost of a GP visit was included.
A&E attendance	138	Applied to all episodes of care.

*given the small number of ambulance transfers identified, we anticipate there are underestimates. GP referrals here are only reflective of those where they are linked to the ambulance transfer and are not reflective of all likely GP attendances which precede an admission

We conducted (multiple) linear regressions (*n*=15 models detailed in Appendix Table 5) to examine variation in the length of stay. We examine heterogeneity and identify if there were significant predictor variables of LOS (using the ‘lm’ function in R). We examined patient-level attributes, disease stage, type, age and gender, and independently evaluated service-level attributes to examine differences in LOS by palliative care exposure. To assess quality outcomes from a patient perspective, we examined admissions in the last 28 days of life, 30-day readmissions and place of death. In patients with two tumours recorded (*n*=19), the total number of care episodes and LOS only were included in analyses.

Finally, hypothesising that expanded use of alternative care models results in reduced use of acute care, we modelled the cost savings by assuming that time patients spend in acute care/hospital could be decreased by either a reduction in the number of UEC admissions (by 5, 10, 15, 20, 25%) or a reduction in the mean LOS in hospital following an admission (by 1, 3 or 5 days) compared to the baseline, as previously deployed in other exemplary reports [26].

## Results

In 2015, 4224 patients died with a recorded cancer diagnosis. Of the patients, 97.9% had complete data for analysis. Of these, 3134 patients (74.1%) were admitted for a total of 60,746 days (averaging 19.5 days) of inpatient UEC during their last year of life; 48.9% (*n*= 1535) had one or more UEC admissions during their last 28 days of life. A total of 6058 episodes of care were delivered. Most cases were admitted to general medicine units (47.6% of first admissions), with the remainder admitted to surgical (12.3%), oncology (5.5%), haematology (2.8%), general assessment (2.3%), urology (1.4%), fractures (1.2%), gastroenterology (1.0%), ENT (0.9%) and cardiology (0.9%) units. We found small numbers of ICU admissions *n*=<10. Intensive rescue activities were found in <1% of cases within the dataset, details shown in Appendix Table 6. The total LOS for ICU-identified cases was 19 days. There were 1008 episodes where patients had readmissions within 30 days (Appendix—“30-day readmissions”).

The total estimated cost to the health service for unscheduled care was £28,684,261, averaging £9200 per person admitted. Inpatient specialist palliative care (support) was identified in 800 records (25.5% of those admitted). Where palliative care input was indicated in an UEC record, the additional cost, as attributed per bed day, contributed £1,322,328 (or 4.6%) of total costs, averaging £1653 per person identified. Ambulance transfers (*n*=89) contributed £21,004 to the total estimated costs, with a further £18,971 in identifiable GP referral costs for those (7.9%) of patients identified as having a preceding GP referral recorded. Additional LOS costs by age, gender, socio-economic status and admissions in the last 28 days of life and place of death are given in Appendix—Fig. 2, “Gender”, “Socio-economic group”, Table 7 and Fig. 3.

### Age

Of those admitted, 0–19-year-olds (*n*<10) had the greatest mean inpatient days (25.3 days, standard deviation (SD) 32.4 days) with mean average costs of £9030, followed by 80–89-year-olds (*n*=649; mean =20.1 days, mean cost = £7855). Third highest mean LOS was in 60–69-year-olds (*n*=782, 19.9 days, mean cost = £7822), followed by 70–79-year-olds (*n*=938, 19.5 days, mean cost = £7718), and 90+-year-olds (*n*=131, mean = 18.9 days, mean cost = £7399) and 40–49-year-olds (*n*=148, 18.7 days, mean cost = £7530). Those aged 20–29 and 50–59 years old (*n*=17 and *n*= 399 respectively) had the lowest mean days (both 17.9 days, mean cost = £7118 and £7189 respectively) with slightly higher mean LOS in 30–39-year-olds (*n*=46, 18.1 days, mean cost = £7213). In line with absolute costs, the largest overall total inpatient days were amongst those aged 70–79 years (see Appendix—Fig. 2).

### Stage of disease

**Fig. 1:**
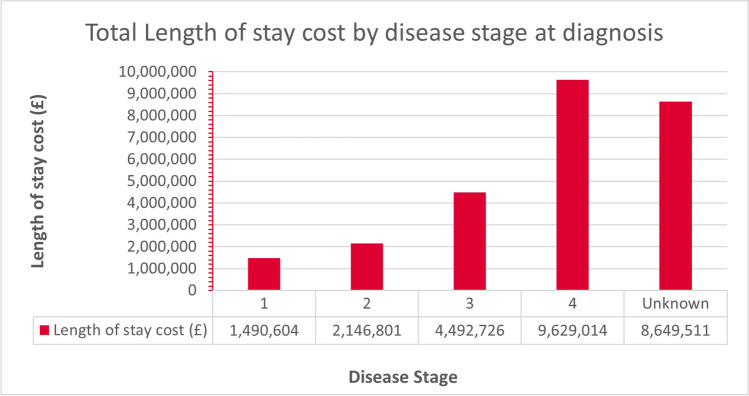
Length of stay costs by disease stage at diagnosis

**Fig. 2:**
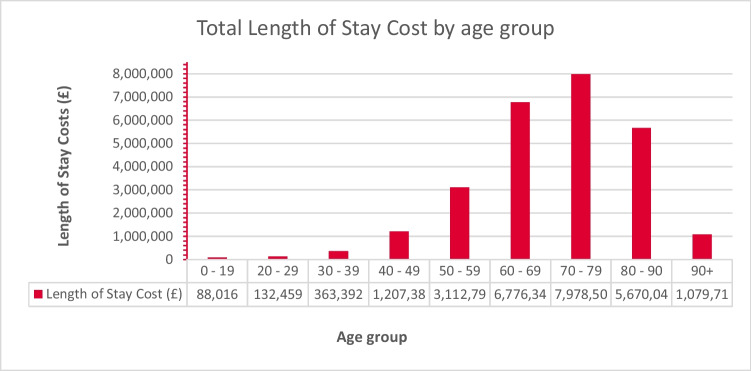
Length of stay cost by age group

**Fig. 3:**
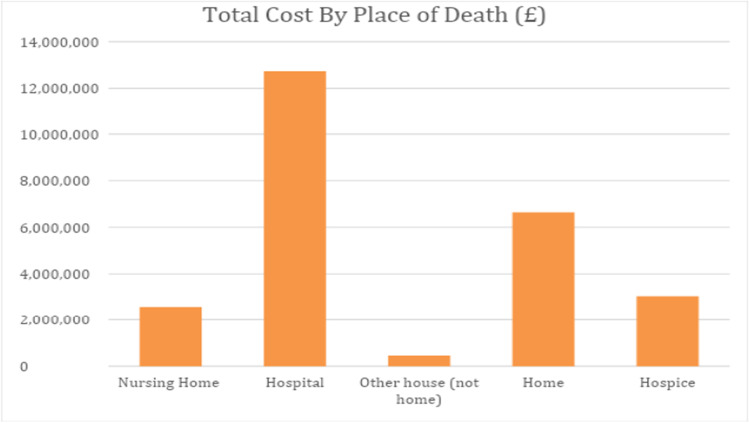
Length of stay cost by place of death

The highest service use and total cost were in those diagnosed with stage IV disease (*n*= 1198; 38.4%), requiring 22,099 days of inpatient UEC, with an attributed LOS cost of £9,629,014 (Fig. 1). Notably, the mean LOS was 18.4 days (mean cost = £7377, SD= £5821), in this group, which was the lowest. Those with an unknown stage at diagnosis had the second greatest utilisation of UEC (*n*=960) which required 19,851 days of UEC, costing £8,649,511 with a mean inpatient stay of 20.7 days. Those who presented with stage III disease (*n*=542) used 10,311 days of UEC, averaging 19.0 days and costing £4,492,726. Those with stage II (*n*=259) used 4927 days of UEC, costing £2,146,801. The lowest overall service use and total costs for UEC in the last year of life were amongst those with stage I disease at diagnosis (*n*=159, used 3421 days UEC). However, the mean LOS was greatest in those with stage I disease, with 21.5 days, with the highest mean cost (£8293, SD= £6866) at an overall cost of £1,490,604.

### Tumour type

Lung cancer patients had the highest proportion of admissions for UEC (23.2%, *n*=788), with a mean LOS of 17.9 days (SD=17, mean cost = £7224, SD= £5418, see Table 2) requiring 14,078 total days of UEC, at a total cost of £6,134,090. The second-highest use was in colorectal and anal cancer patients (11.4%, *n*= 364), requiring 6901 days of UEC at a total cost of £3,006,915, with a mean LOS of 19 days (SD=17, mean cost = £7565, SD= £5700), followed by oesophageal and stomach cancer patients (7.1%, *n*=238) 4295 days of UEC at a total cost of £1,871,425 (mean LOS = 18 days, mean cost pp = £7300, SD= £5079). Full details by cancer type are shown in Table 2. The mean inpatient days (for UEC) were greatest in small intestine cancer patients (30.3 days), and lowest in non-melanoma skin cancer patients (NMSC, 10.9 days), with parallel highest mean cost in small intestine cancers (£10,807) and lowest mean cost in those with NMSC (£4846).

**Table 2:** Costs and inpatient days by disease type

Cost per patient (£)								Inpatient days					
	*n*	%	Mean	Standard deviation	Min	Max	Total attributed LOS cost	Total inpatient days	% of total inpatient days	Mean	Standard deviation	Min	Max
Brain	83	2.7	7587	5635	1609	32,328	688,440	1580	2.6	19	16	1	73
Breast	194	6.2	6658	4530	1609	28,962	1,371,652	3148	5.2	16	14	1	69
Colorectal & Anus	364	11.7	7565	5700	1609	42,307	3,006,915	6901	11.4	19	17	1	134
Connective Tissue	11	0.4	9281	7460	1609	24,372	115,466	265	0.4	24	21	1	66
Female Genital ex ovarian	59	1.9	7649	5754	1609	31,597	513,280	1178	1.9	20	19	1	99
Head & Neck	88	2.8	9430	10,721	1609	71,768	939,852	2157	3.6	25	30	1	170
Leukaemia	102	3.3	7674	6047	1609	31,597	898,022	2061	3.4	20	19	1	99
Liver, Gallbladder & bile ducts	129	4.1	7470	5266	1609	32,141	1,055,754	2423	4.0	19	16	1	86
Lung	788	25.3	7224	5418	1609	69,541	6,134,090	14,078	23.2	18	17	1	223
Lymphoma	123	3.9	10,062	8256	1609	48,733	1,504,547	3453	5.7	28	26	1	155
Melanoma	32	1.0	6466	3900	1609	18,133	222,218	510	0.8	16	12	1	55
Mesothelioma	33	1.1	6532	3930	1609	18,201	229,625	527	0.9	16	11	1	42
Myeloma	72	2.3	9316	6620	1609	41,389	782,556	1796	3.0	25	21	1	131
NMSC	16	0.5	4846	3171	1609	11,690	75,816	174	0.3	11	10	1	32
Oesophagus & Stomach	238	7.6	7300	5079	1609	28,673	1,871,425	4295	7.1	18	16	1	84
Other—small numbers	22	0.7	8669	7600	1609	34,963	221,782	509	0.8	23	25	1	110
Other digestive	27	0.9	7990	5082	1609	21,975	236,597	543	0.9	20	15	1	62
Other female genital & fallopian tube	13	0.4	8620	5938	1609	20,275	126,795	291	0.5	22	19	1	62
Ovarian	65	2.1	7748	5727	1609	32,957	566,002	1299	2.1	20	17	1	77
Pancreas	170	5.5	7164	4808	1609	25,528	1,310,215	3007	5.0	18	14	1	64
Prostate	189	6.1	8024	6831	1609	47,203	1,691,907	3883	6.4	21	19	1	108
Retro peritoneum	12	0.4	9317	6228	1609	19,357	119,823	275	0.5	23	18	1	59
Small intestine	12	0.4	10,807	8045	2153	28,537	158,167	363	0.6	30	27	2	89
Unknown primary	104	3.3	8409	6662	1609	51,487	969,916	2226	3.7	21	20	1	164
Urinary cancer	172	5.5	8232	5604	1609	39,332	1,597,791	3667	6.1	21	17	1	113

Table shows the average costs and inpatient days by disease of origin, excluding those where two tumours were recorded. Total costs attributed by inpatient length of stay (LOS) and proportion of all inpatient stays provided. Costs are reported by 2015 index costings. Where rare cancers which had numbers below appropriate disclosure levels were reported, these were collapsed into one group and reported here as ‘other—small numbers’ to ensure the data were included for comparison

### Palliative care as a factor influencing length of stay

Specialist palliative care support was recorded as provided to 800 patients during admissions in their last year of life, requiring 11,112 days of UEC provision, costing £1,322,328. The mean cost of care for those who did not receive palliative care was £6959, and £9849 for those who did. We compared three observed variations of palliative care support received as a potential indicator of efficacy, in those (a) with further admissions noted afterwards, (b) who died before discharge and (c) with no subsequent admissions.

Linear regression of differences in LOS using those with no palliative care as the reference value showed relative differences between coefficients in the groups (Table 3). Relative to the intercept (18.4), group A (with further admissions) had 9.7 additional days of UEC, group B (those who died before discharge) had 3.2 additional days and those in group C (with no further admissions) had −0.8 days relative to those with no palliative care recorded during admissions.

**Table 3:** Costs and length of stay by palliative care status

Cost (£) pp									Inpatient days			Relative difference in average LOS
	*n*	Mean	Standard deviation	Min	Q1	Median	Q3	Max	Mean length of stay (LOS)	Standard deviation	Coefficient (linear regression)	Overall mean LOS = 19.5
*Palliative care status*												
No	2318	6959	5403	1609	3139	5587	8953	69,541	18.5	17.7	18.5	−1.0
Yes—with further admissions after noted	251	11,636	6418	2034	7109	9905	15,031	39,332	28.2	18.3	9.7	8.7
Yes—last admission & died before discharge	250	8203	5630	1728	4474	7134	10,398	45,758	21.7	21.3	3.2	2.2
Yes—with no further admissions after noted	299	9707	8156	1728	4703	7678	11,839	71,768	17.7	14.8	−0.8	−1.8

Regression analyses compared costs and length of stay by the patient-level use of palliative care services during their admissions, where patients were stratified by their index use of palliative services during admission and then categorised to understand if their future care outcomes were mediated by their access to palliative services

### Attributes affecting length of stay

Iterative addition of patient-level characteristics and service variables (age, stage, gender, palliative care input, place of death) were examined by regression to estimate the variability in LOS (detailed in Appendix, Table 5). In primary analyses, which included all cases including those not admitted for care, up to 41% of the variability in LOS was accounted for (a maximum of 14 variables included). When restricted to only those who received UEC, only 15% of the variability of the LOS could be accounted for.

### Potential impact of reduction in emergency admissions or length of stay

From the baseline of £28,684,281 total costs and a mean LOS of 19.5 days, a reduction of 1 day in mean LOS without any additional reduction in the overall number of admissions would release £1,520,267 (Table 4). On average, a 3-day reduction of mean LOS, combined with a 10% reduction in admissions, implies £7.37 million reductions in the UEC cost in the last year of life. The maximum reduction hypothesised, with a 5-day reduction of mean LOS accompanied by a 25% reduction in admissions, would potentially release £14.71 million in UEC costs.

**Table 4:** Potential release of resources compared to the baseline unscheduled care costs in the cohort of cancer patients in their last year of life (£)

Reduction in mean LOS (days)				
Reduction in % of emergency admissions	0	1	3	5
0	0	1,519,405	4,500,879	7,539,689
5	1,433,401	2,952,806	5,934,280	8,973,090
10	2,866,802	4,386,207	7,367,681	10,406,491
15	4,300,203	5,819,608	8,801,082	11,839,892
20	5,733,604	7,253,009	10,234,483	13,273,293
25	7,167,005	8,686,410	11,667,884	14,706,694

Quoted in £, based on the costs estimated for the entire cohort of cancer patients. Following the methods of Hatziandreu et al. [26], the overall cost of care in the last year of life (~£28m) was subjected to hypothetical reductions in mean length of stay, and then further reductions were hypothesised should the overall volume (%) of admissions occur through policy or practice changes

## Discussion

To the best of our knowledge, our results provide the first estimates of the cost consequences of unscheduled emergency care admissions in cancer patients in the last year of life in Northern Ireland, showing that ~three in four patients required admission during the last year of life, averaging 19.5 days of care. More than 60,000 days of unscheduled emergency care were delivered. Concerningly almost half of those patients experienced unscheduled inpatient care during the last 28 days of life.

Despite a substantive cost burden of ~ £28 million, less than 5% of costs were contributed by specialist inpatient palliative care input. Data showed a minority of patients (25.5% of those admitted) accessed palliative care, which is significantly lower than other reports, where up to 64.5% of cancer patients had at least one palliative care event [27]. Although individual patients may have displaced activity in other sectors, the level of access or availability in our cohort remains concerning, considering the high proportion of deaths in hospitals and the high numbers accessing care in the last 28 days of life. These results highlight the need for priority action since evidence suggests specialist palliative and end-of-life care can reduce hospital admissions [22, 28], and could reduce the likelihood of costly and aggressive treatments in the last days of life, where patients can access palliative care at an early stage [27].

More than one in three of those admitted was diagnosed with stage four disease, which required £9.6 million in UEC costs. In keeping with global trends [29], the largest proportion of patients (one in four) admitted had lung cancer, and are understood to be at risk of later diagnosis (67.8% likely to be diagnosed at an advanced stage [30]). Whilst these patients had a lower mean LOS (17.9 days) than the overall average, their total attributable cost was greatest (~£6 million). Thus, prompt consideration of the return on investments in lung health checks [31] should be prioritised. Importantly, recent supportive trial data on low-dose CT screening for early diagnosis of lung cancer indicate mortality benefits [32]. Attendant ‘time to benefit’ estimates remain several years; thus, prompt consideration of building appropriate capacity is vital, due to costs associated with the status quo of late diagnosis of potentially avoidable cancers.

Concerningly, patients with colorectal cancers were the second-highest users of UEC, costing more than £3 million; efforts to redistribute stage and refine outcomes for this group should be priority policy decisions. In light of the significant morbidity and mortality in this disease [33], where there are existing screening programmes for early diagnosis, expanded access to the screening process with more effective disease detection testing thresholds might improve patient outcomes [34] and prompt reductions in UEC.

Although those 0–19 years had the highest average days and costs of care, those aged 70–79 years had the greatest total costs of care (£7.9 million). Therefore, targeting the LOS in children and young adults may not leverage the same benefit as intervening in those aged 70–79 years. Our result concurs with a recent valuation of informal care to cancer decedents at end of life which showed that the mean age of recipients was 70–79 years [35]. This study identified the monetary value of informal care at £948.86 per week, derived from the relative costs at the wage rates of those who might otherwise complete the care (the proxy good method). They report that care was in the majority (65%) delivered by females, 55% of whom were previously in employment. The implications identify the significant shortfall in the replacement cost of their care relative to Carers’ Allowance payments (not including lost income and collateral costs on productivity in this valuation). The attendant implications for our study highlight the potential for displacement effects shown by our results, where informal carers were unable to meet their needs. Many cannot afford or indeed may be supported by employers to adopt a carer role in the order of the hours reported (93 h per week) [35]. Thus, some of those 1535 people in our study who sought care in the last 28 days of life may reflect choices that stem from practical considerations and difficulties in hospice care access. The next appropriate step for this cohort would be to match data on primary care and scheduled care use, staffing ratios and detail on formal or informal carer access, to examine if age-based use of services is mediated by these factors rather than the disease characteristics, co-morbidity [36] or palliative and hospice care provisions.

Finally, the paucity of data on inpatient care constrained the characterisation of (quality) markers of aggressive care or palliative care at the end of life provided, as drivers of LOS. Although regression models explained 41% of LOS variation, evidently, factors not included in the data greatly influence LOS. Whilst staffing ratios may be a factor [37], their attributable weighting is unclear. Moreover, many administrative datasets do not provide standardised reporting; therefore, minimum data sets and indicator development should seek to harmonize across jurisdictions [38], since a review of palliative care costing in the UK [39] suggested lacking data has implications on how palliative care (and palliative care research) is planned and funded. Thus, developing accurate coding for palliative care services, accurately mapped to patient-centred outcomes and factors attributed to higher levels of congruence in preferred place of care [40], are essential to ensure critical insights and evaluations are possible to enable international benchmarking and to redress inequity and improve patient experiences at the end of life.

## Limitations

Recognising service displacement, future work should consolidate healthcare use trajectories reflecting the sequence of patient episode data (primary and planned care) to complement the insights described. However, a review of palliative care costs showed that hospital costs accounted for the greatest proportion of the overall cost of care [39]. We are therefore reassured to have prioritised the area most likely to reflect the greatest overall costs, and thereby, trends herein are likely to convey greater relevance in clinical optimisation decision-making.

Within the administrative admissions dataset, it was impossible to distinguish between acceptable interventions and potentially unnecessary ‘rescue’ care, but there were low levels of intensive activities overall. Critically, with the data available, it was not possible to identify how many days within an episode palliative care was contacted; therefore, it was not possible to fully characterise (specialist palliative) care input. Necessarily, our approach will result in a lower estimated level of gross expenditure than the real-world scenario but may result in an overestimate of absolute palliative care days provided. Thus, relative differences in LOS cannot be used to determine palliative care efficacy and are exploratory only.

## Conclusions

Unscheduled care use in the last year of life of cancer patients poses a significant time burden for patients costing acute health services ~ £28 million. Existing data infrastructure limits the ability to fully characterise causality and quality aspects of this care. Opportunities to focus service reconfiguration and early detection efforts for those with the highest cost use, prioritised in lung and colorectal cancers, would provide the greatest potential to reduce care burdens.

## Appendix

### 30-day readmissions

In total, there were 1008 episodes where readmission within 30 days of a previous admission occurred (1.65% of all admissions), with 226 individuals having multiple episodes of 30-day readmissions during their last year of life. Although not directly applied in the final cost model, we calculated a hypothetical readmission ‘penalty’ or tariff incurred, as a marginal difference between one additional day of excess bed day costs on the LOS of the previous admission and the cost of a new admission Day 1 cost, which was equal to £1303 per episode. Thus, for these (1008) events, where patients were readmitted on days 2-30 after a prior admission, the consequent hypothetical additional UEC cost ‘penalty’ of these 30-day readmissions was £1,313,424. This amount reflects a minimum cost since it does not reflect the duration of the readmission LOS.

**Table 5:** Summary of all regression analyses

	Adjusted *R*-squared		
	Variables analysed to examine variability in length of stay	All deaths	In admitted cases only
Model 1	Gender	0.0010	
Model 2	Place of death	0.0336	
Model_2b	Survival time	0.0003	
Model 3	ICD 10 group	0.0133	
Model 4	Stage	−0.0005	
Model 5	Age at diagnosis	0.0011	
Model 6	Age at diagnosis + SES	−0.0001	
Model 7	Gender + ICD 10 group + stage + age + SES	0.0146	
Model 8	Gender + ICD 10 group + stage + age + SES + place of death	0.0449	
Model 9	Gender + ICD 10 group + stage + age + SES + place of death + palliative care	0.2675	
Model 10	Gender + ICD 10 group + stage + age + SES + place of death + palliative care + first hospital admission location	0.2848	
Model 11	Gender + ICD 10 group + stage + age + SES + place of death + palliative care + first primary admission diagnosis + age at death	0.3551	
Model 12	ICD 10 group + stage + age + SES + place of death + palliative care + first primary admission diagnosis + age at death + first hospital admission location	0.3765	
Model 13	Gender + ICD 10 group + stage + age + SES + place of death + palliative care + first primary admission diagnosis + age at death + first hospital admission location	0.3767	
Model 14	Gender + ICD 10 group + stage + age + SES + place of death + palliative care + first primary admission diagnosis + age at death + first hospital admission location + settlement + last admission primary diagnosis	0.4127	0.1526
Model 15	Gender + ICD 10 group + stage + age + SES + place of death + palliative care + first primary admission diagnosis + age at death + first hospital admission location + settlement + last admission primary diagnosis + survival time	0.4126	0.1522

Note: using the ‘lm’ function in R, with each regression variables were added iteratively to examine a series of data variables thought to potentially mediate length of stay. Each model of the latter models was examined for both all patients in the dataset and then separately for only those with an admission. *SES* = socio-economic status

**Table 6:** Frequency of OPCS-4 codes for assisted ventilation and other intensive outcomes in the linked hospitalisation data

Outcome	OPCS code	Description	Frequency of reports in the dataset
Use of CPAP	E85.6	Continuous positive airway pressure	0
Use of NIV	E85.2	Non-invasive ventilation NEC	21
Use IMV	E85.1	Invasive ventilation	<10
Use of ECMO	X58.1	Extracorporeal membrane oxygenation	<10
Use of renal replacement therapy	X40.1	Renal dialysis	0
	X40.3	Haemodialysis NEC	<10
	X40.4	Haemofiltration	<10
Total			40

Note: Given the small numbers of ICU admissions *n*=<10, we sought OPCS-4 codes to characterise intensive rescue activities within episode data to examine these as drivers of LOS. <1% of cases identified these procedures; not all were indicated to have been admitted to ICU. Total LOS for ICU-identified cases = 19 days, and therefore ICU was unlikely to be a driver of LOS

### Gender

Of those accessing UEC, the minority were female (n=1394) compared with (n=1740) male. Whilst a higher proportion of males (75.7%) had at least one emergency admission recorded in the last year of life compared to females (72.5%; p=0.018), the mean LOS was similar - for females 19.2 days compared with 19.6 days for males (accounting for 44.1% and 55.3% of total inpatient days respectively). The total cost for UEC in the last year of life was £11,638,126 for females and £14,605,391 for males. The total LOS for females (26,710 days) was lower than for males (33,520 days). Mean costs for females £7,632 (SD = £5,490) was similar to males (£7,720, SD= £6313).

### Socio-economic group

Although there were no statistically significant differences by socio-economic groups, there were greater numbers of people admitted for UEC in the last year of life from the most deprived socio-economic group (SES5 n=680, 21.6%, total cost £5,691,397, Table 7) as compared to the least deprived (SES1) n=586 (total cost £4,896,205). In SES2 (n=643) total UEC cost was £5,395,542), SES3 (n=605) total UEC cost was £5,335,848, SES4 (n=599) total UEC cost was £5,069,622. However, the mean UEC cost was highest in SES3 (£7,975) while the lowest average UEC cost was in SES2 (£7,516).

**Table 7:** Costs and length of stay by socio-economic groups

Socio-economic status group	Number	Mean cost (£) per person	Standard deviation	Mean inpatient days	Standard deviation	Total inpatient days (%)	Costs (£)
SES1 (least deprived)	586	7547	5237	19.2	15.8	11,237 (18.5%)	4,896,205
SES2	643	7516	5967	19.3	18.7	12,383 (20.4%)	5,395,542
SES3	605	7975	6259	20.2	19.5	12,246 (20.2%)	5,335,848
SES4	599	7624	5316	19.4	16.3	11,635 (19.2%)	5,069,622
SES5 (most deprived)	680	7738	6712	19.2	19.1	13,062 (21.6%)	5,691,397

Note: Costs based on length of stay costs only. Socio-economic status was based on the postcode of residence at the time of death. Super output areas (SOA) were assigned to each patient based on their postcode of usual residence at the time of death. The patient was then assigned, through their SOA, a socio-economic deprivation quintile based on the SOA’s 2017 income domain of the Multiple Deprivation Measure. The 2017 Multiple Deprivation Measure is available from the NI Statistics and Research Agency (www.nisra.gov.uk)

### Admissions in the last 28 days of Life & Place of Death

Admissions in the last 28 days of life and place of death. Of those (*n*=3134) people admitted during the last year of life, we found *n*=1535 patients had admissions in the last 28 days of life. For this, we total the final admission length of stay for each person. The final admissions, where the admission is noted to have occurred within the last 28 days of life, require a total of 18,638 days of unscheduled care. Based on this, we estimate the cost of unscheduled care delivered to those within the last 28 days of life was £7,694,212 (~ 26.8% of total costs, mean cost pp = £5012). Of those who had an admission within the last 28 days of life, *n*=371 (24.2%) had received palliative care within one or more of their unscheduled care episodes, and *n*=530 people (34.5%) died before discharge. Comparing within those (*n*=530) people who died before discharge, 71.5% did not have palliative care involvement documented in their unscheduled care record (*n*=379 people). An analysis of the data by the place of death shows that those who went on to die in hospice care (*n*=455) had the greatest average time from diagnosis to death (927 days) and the lowest average UEC cost in the last year of life £6680 per person; total cost in this group was £3,039,381 (Fig 3). Those who went on to die in nursing home care (*n*=270) had an average time from diagnosis to death of 814 days and had the highest average cost of UEC in the last year of life £9555 per person, total cost £2,579,831 (note here we do not explicitly know if the persons were ordinary residents in nursing homes pre-/during and post-diagnosis). Those who went on to die at home (*n*=966) had the shortest average time from diagnosis to death of 739 days, average cost £6876 and total cost £6,642,295. In those who died in hospital (*n*=1494, 47% of those with UEC episodes), the average time from diagnosis to death was 752 days, with total costs of £12,733,465, with an average cost of £8523 in their final year of life.
